# Protocol of a Series of *N*-of-1 trials for Exploring Personalized Blood Glucose Responses to Different Staple Foods in Patients with Diabetes

**DOI:** 10.1016/j.cdnut.2025.107575

**Published:** 2025-10-13

**Authors:** Yuyang Wang, Yuexiao Chen, Cheng Li, Juanjuan Duan, Yaqiong Guo, Shuai Zhu, Baoming Li, Defu Ma, Jing Zhu

**Affiliations:** 1 Institute of Biotechnology and Health, Beijing Academy of Science and Technology, Beijing, China; 2 Department of Social Medicine and Health Education, School of Public Health, Peking University Health Science Center, Beijing, China

**Keywords:** N-of-1 trial, type 2 diabetes mellitus, staple food, postprandial blood glucose responses, personalized nutrition

## Abstract

**Background:**

Effective control of postprandial glucose responses (PPGRs) is essential for managing the progression of type 2 diabetes mellitus (T2DM). Carbohydrate-rich staple foods are known to induce significant variability in PPGR among individuals.

**Objectives:**

This study aimed to investigate the personalized PPGR to various staple foods among patients with diabetes using an *N*-of-1 trial design.

**Methods:**

A single-center, randomized, crossover *N*-of-1 trial is planned. Participants will receive 5 different staple foods (white rice, germ rice, brown rice, rice noodles, and pasta) 3 times in a randomized order. Continuous glucose monitoring will track PPGR at 5-min intervals. The primary outcome will be the postprandial blood glucose peak. The Bayesian analysis will be conducted at both individual and group levels to access the PPGR for staple foods.

**Discussion:**

By using the *N*-of-1 trial methodology, this study is anticipated to reveal substantial interindividual variations in PPGR and to identify staple foods that are most effective in stabilizing blood glucose concentrations for individual patients, providing insights into personalized nutritional management for T2DM.

This trial was registered at Chinese Clinical Trial Register as ChiCTR2400090138 (24 September, 2024).

## Introduction

According to the International Diabetes Federation, in 2021, ∼540 million individuals aged 20–74 y were living with diabetes worldwide, and this number is expected to rise to 640 million by 2030 [1,2]. Consequently, diabetes constitutes a substantial disease burden and economic loss globally, underscoring its prevention and management as a critical international public health priority.

Postprandial hyperglycemia has been identified as a key factor in the aggravation and progression of diabetes [3]. Dietary components, particularly the quality and type of staple foods, are significant determinants of blood glucose concentrations and thus play an extremely critical role in the development and management of type 2 diabetes mellitus (T2DM) [4]. Staple foods, which are the primary sources of carbohydrates and energy in the diet, directly impact blood glucose concentrations. Typical staple foods include grains (e.g., rice and wheat) and tubers (e.g., potatoes), with rice constituting ∼70% of the daily caloric intake in developing countries in Asia [5]. The glycemic index (GI) is a metric that quantifies the impact of dietary carbohydrates on blood glucose concentrations, indicating the rate and extent of their digestion, absorption, and subsequent entry into the bloodstream. Recent research has identified several staple foods with a lower GI or other characteristics that can attenuate postprandial glucose responses (PPGRs), including pea noodles and oat noodles [6,7]. It is important to acknowledge that these findings pertain to the intrinsic properties of the foods consumed.

However, glycemic responses to identical foods exhibit substantial interindividual heterogeneity, with significant intraindividual variations even across different contexts [8]. It is noteworthy that such variations exist equally in both diabetic and nondiabetic individuals, which undermines the practical utility of standardized dietary guidelines [9]. Sayegh et al. [10] found that among subjects consuming the same test meal, the variability in PPGR ranged from 25% to 56% both between individuals and within the same individual. This variability may originate from 3 interrelated domains: biological characteristics, food properties, and behavioral contexts. Physiologically, insulin resistance delays the clearance of glucose, whereas the composition of the gut microbiota regulates the rate of carbohydrate fermentation and the efficiency of glucose absorption [11]. Studies have shown that the abundance of *Prevotella copri* is negatively correlated with the SD of blood glucose concentrations [12]. Genetic variations in glucose transporters (e.g., *SLC2A2*) further contribute to different metabolic responses [13]. Refined grains, which lack the fiber that slows digestion, and cooking methods (e.g., overcooking rice) alter the starch structure, increasing their glycemic potential [14]. Meanwhile, behavioral factors such as eating quickly or chronic stress exacerbate blood glucose fluctuations by disrupting satiety signals and insulin sensitivity [15]. These findings highlight the limitations of using average group GI values to predict individual glycemic responses. Consequently, for patients with diabetes, the selection of staple foods that align with individual needs is essential for managing PPGR. Personalized nutrition, which emphasizes the importance of customization based on individual differences, involves tailored dietary recommendations and nutritional plans that consider specific needs, physiological characteristics, genetic backgrounds, health conditions, and lifestyles [16,17].

Single-case randomized controlled trials, also known as *N*-of-1 trials, provide novel insights into personalized nutrition research [18]. These trials determine individual responses to specific interventions through prospective, randomized crossover designs conducted either on individual patients or a series of patients [19]. Previous studies have used *N*-of-1 trials to explore individual differences in PPGR to diets with varying macronutrient compositions in healthy adults [20]. However, the personalized differences in PPGR to various staple foods among patients with diabetes remain understudied.

The present study uses *N*-of-1 trials to investigate the personalized differences in PPGR to different staple foods in patients with diabetes, thereby providing new insights and evidence for personalized nutritional interventions in diabetes management.

## Methods

### Study design

A randomized, 3-period crossover *N*-of-1 trial will be conducted to assess the individual variation in the consumption of staple foods.

### Setting

The study will be conducted at the Institute of Biotechnology and Health, Beijing Academy of Science and Technology in Beijing. The study has obtained ethical approval from the Inner Mongolia Nutrition Society Ethics Committee (NO.A202309002) and is registered with the Chinese Clinical Trial Register (ChiCTR2400090138).

### Study population

Participants will be recruited from communities through advertisements posted on community bulletin boards in Beijing. The inclusion criteria of the participants are as follows: *1*) age between 18 and 70 y; *2*) T2DM diagnosis by an endocrinologist for ≥3 mo; *3*) currently under lifestyle intervention alone or stable single hypoglycemic drug treatment; *4*) be able to operate a smartphone; and *5*) be able to provide written informed consent. The exclusion criteria include the following: *1*) pregnancy, breastfeeding, or plans to become pregnant; *2*) severe metabolic disorders, gastrointestinal diseases, or psychiatric conditions; *3*) use of antibiotics within the past month prior to enrollment; *4*) adherence to special dietary patterns (e.g., ketogenic diet and low-carbohydrate diet); *5*) difficulty with chewing foods; *6*) a history of allergies or intolerance to the test foods; and *7*) frequent travelers.

Participants will maintain daily medication logs, and the research team will verify adherence through telephone follow-ups. Any adjustments to medication during the trial will result in the participant’s exclusion. If the person is interested in the study, the study investigator will schedule a personal online or a face-to-face meeting to provide oral information and answer questions. Prospective participants will be informed that they have ≥24 h to consider their participation in the study. Should they choose to participate, both the participant and the study investigator will sign the written informed consent, after which the investigator will conduct the screening process. If all inclusion criteria are fulfilled, and none of the exclusion criteria are met, the person will be included in the study. Participants have the right to withdraw from the *N*-of-1 trial at any time. All instances of participant dropout or trial interruption will be documented, including the time and reason for discontinuation.

### Informed consent statement

Informed consent was obtained from all participants and/or their legal guardians before participating in this study. This study will be conducted in accordance with the Declaration of Helsinki.

### Test diets

The staple foods were cooked, and the main nutritional compositions of cooked (ready-to-eat) staple foods were analyzed by the Beijing Institute of Nutritional Resources, with the results presented in Table 1. To enhance the participants’ dining experience and contextualize the staple food within a typical breakfast setting, 5 test diets will be designed: white rice, germ rice, brown rice, rice noodles, and pasta. The variable component of the test diet is staple food; all other elements remain consistent. Each test diet includes a staple food providing 50 g of available carbohydrates, alongside 150 g of scrambled eggs with tomatoes, 50 g of cucumber, and 200 mL of milk. The detailed menus of each test diet can be found in Table 2.

**TABLE 1:** Nutritional composition of 5 test staple foods in the *N*-of-1 trials (g/100 g)^1^.

Test subject	Protein	Fat	Total carbohydrates	Total dietary fiber	Available carbohydrates
Refined rice	2.80	0.2	35.4	0.378	35.0
Brown rice	2.89	1.0	31.7	2.830	28.9
Germ rice	2.38	0.5	29.1	0.474	28.6
Rice noodles	5.24	0.5	28.6	3.35	25.3
Pasta	3.69	0.4	29.6	1.540	28.1

1 The values were determined based on the cooked state (ready-to-eat).

**TABLE 2:** A sample staple menu of the first cycle (period 1) of the *N*-of-1 trial^1^.

Food items	Test diet A	Test diet B	Test diet C	Test diet D	Test diet E
Refined rice (g)	143.0	—	—	—	—
Brown rice (g)	—	173.0	—	—	—
Germ rice (g)	—	—	175.0	—	—
Rice noodles (g)	—	—	—	198.0	—
Pasta (g)	—	—	—	—	178.0
Stir-fried tomato and eggs (g)	150.0	150.0	150.0	150.0	150.0
Cucumber (g)	50.0	50.0	50.0	50.0	50.0
Milk (mL)	200.0	200.0	200.0	200.0	200.0

1 Each subject was assigned a randomized staple food menu across the 3 cycles (sets) of the *N*-of-1 trials. The available carbohydrate content for each staple food was standardized to 50 g, and the weights of the staple foods for the 6 interventions were prepared accordingly. Each staple food intervention included the same weight of side dishes (scrambled eggs with tomatoes, cucumber) and milk to ensure balanced dietary intake.

### Study procedures

**FIGURE 1:**
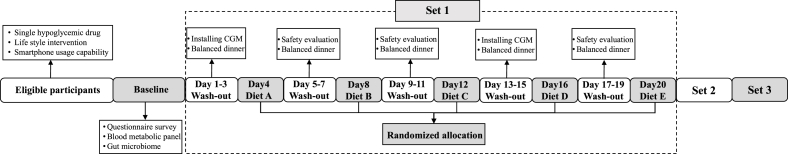
Flowchart of *N*-of-1 trials for personalized differences in staple foods with diabetes. (Except that the 5 test diets are administered in an independently generated randomized sequence within each period, the other study procedures across the 3 periods, such as continuous glucose monitoring, safety evaluation, duration of the washout period, and provision of balanced dinners, are identical.) CGM, continuous glucose monitoring.

The study scheme is outlined in Figure 1. The study consists of 3 periods, each of which comprised 5 test days. On each test day, a different test diet will be consumed for breakfast. Within each period, the 5 test diets will be administered in a randomized sequence. By referring to the washout period duration (48 h) in previous studies by Zavitsanou et al. [21] and considering that key indicators such as postprandial blood glucose-related parameters can return to baseline concentrations within 2–3 d (thus effectively eliminating the residual effects of the dietary used in the previous test), a 3-d washout period was set between each test in this study. During the washout period, subjects were instructed to maintain their regular dietary habits [21,22]. To ensure consistency across test diets and to minimize the impact of prior dietary intake, participants will be advised to restrict their consumption of high-protein, high-fat, and high-fiber foods on the day preceding the test day. Additionally, they will be provided with a balanced dinner on the evening before the test day and will be required to fast for 12 h prior to the test diet. They will also be instructed to avoid strenuous physical activity and alcohol consumption on the day before the test day, with this requirement reinforced through both in-person and WeChat-based instructions. Except for the differences in the random allocation sequence of the 5 test diets, the other study procedures across the 3 periods are identical, such as blood glucose monitoring, safety evaluation, duration of the washout period, and provision of balanced dinners.

On the test day, participants will be required to rest for ≥10 min prior to the serving of the test diet. The test meal will be prepared and served at the Beijing Institute of Nutritional Resources restaurant across all participants. The cooking of each staple food follows strictly standardized preset procedures, using a fixed staple-to-water ratio and cooking time. For white rice, brown rice, and germ rice, the ratio of staple food (grams) to water (milliliters) is 1:1.25, and they will be cooked for 40 min using a Supor SY-60YC8001Q rice cooker (Zhejiang Supor Co, Ltd.). For rice noodles and pasta, the mass-to-volume ratio of staple food (grams) to water (milliliters) will be set to 1:10 to ensure that the rice noodles or pasta ensure complete submersion and uniform heating throughout the cooking process. They will be boiled for 10 min, after which the excess water will be drained thoroughly before being incorporated in the test diet. The time of the first bite will be synchronized with the glucose monitor intervals and recorded for each participant. The participants will be directed to refrain from consuming any additional food or to avoid engaging in strenuous physical activity for ≥3 h following the consumption of the test diet.

### Randomization

The randomization process for the trial will be conducted by a statistician who is not involved in the data analysis to ensure impartiality. Allocation concealment will be achieved using sequentially numbered, opaque, sealed envelopes. The test diets will be designed in alphabetical letters. A random sequence will be generated using R software (R Foundation), with each participant’s order being independently randomized for each period. The order will be provided directly to the food preparation staff, who have no involvement in data collection and analysis. This study strictly implemented analyst blinding. Specifically, the statisticians responsible for data analysis remained completely unaware of which specific staple foods the letter codes represented until the final analysis was completed, and the results were locked.

### Sample collection

Following enrollment, participants will be required to provide fasting blood and stool samples during the run-in period to facilitate subsequent biochemical analyses. The collected biological samples will be stored at −80 °C. Fasting blood will be used to test for lipids (total cholesterol, triglycerides, HDL cholesterol, LDL cholesterol, hemoglobin A1c, fasting blood glucose, serum antiglutamic acid decarboxylase antibodies), as well as liver and kidney health markers. Metagenomic sequencing technology will be used to analyze the gut microbiota, providing information on the microbial abundance at various taxonomic levels (phylum, class, order, family, genus, and species) for each sample. Fecal sample DNA will be extracted using the MagPure Stool DNA KF Kit B (Magen Biotech), strictly following the manufacturer’s instructions. Subsequently, metagenomic sequencing of the participants’ fecal DNA samples will be performed using the Illumina HiSeq X sequencing platform (Illumina Inc). The data will undergo quality control using Fastp (v0.20.1) software. Kraken2 will be used for the taxonomic classification of all valid sequences across the samples. MetaGeneMark will be used for gene prediction from the assembled sequences, and the predicted genes will be annotated using commonly used functional databases.

### Data collection

After enrollment, participants undergo anthropometric measurements, including measurements of weight, height, waist circumference, and hip circumference, with the average of 3 measurements being used as the final values. The blood glucose concentrations during the 3 periods will be monitored through a continuous glucose monitoring (CGM) system (Sibionics Technology Co, Ltd) every 5 min. A designated study personnel member who is not involved in the data analysis will be responsible for monitoring and extracting data from the CGM terminal platform (Sibionics Technology Co, Ltd), removing all information capable of identifying participants before forwarding data to statistical analysis to ensure anonymity.

### Outcomes

The primary outcome is the postprandial blood glucose peak (PBGP) within 3 h after the first bite of the test diet. The secondary outcome is the postprandial 3-h glucose incremental AUC (iAUC_3h_), calculated by assessing the change in blood glucose concentrations from the fasting state (measured 30 min before test diet consumption) over the 3-h period following the intake of the test diet.

### Sample size estimation

In this study, the primary outcome, PBGP, was reported at the population level using a Bayesian hierarchical model. Given the absence of formulas for sample size calculation in this type of population design, the sample size calculation in this study referred to the simulation method based on the Bayesian hierarchical model conducted by Stunnenberg et al. [23]. Additionally, the statistical power under different sample sizes was evaluated via the Markov Chain Monte Carlo (MCMC) method. The minimum clinically important difference (MCID) is an indicator used to assess the clinical significance of changes in medical outcomes [24]. It represents the smallest change in clinical outcomes that is considered meaningful by patients, clinicians, or researchers. Referring to the study by Tian et al. [25], the MCID of PBGP in this study was defined as 0.78 mmol/L, which was determined based on a 20% difference in PBGP between patients with T2DM and those with impaired fasting glucose, i.e., 3.89 mmol/L [26]. According to previous studies, the difference in PBGP between patients with diabetes consuming white rice and pasta was 2.4 mmol/L, with a between-individual SD of 2.5 mmol/L and a within-individual SD of 1.2 mmol/L [21]. At the individual level, it was assumed that the true intervention effect of each participant followed a normal distribution; at the observation level, the within-individual variation was set to be the same. Observational data for each subject were randomly generated through simulation involving 3 intervention cycles and 5 staple foods, and a Bayesian hierarchical model was constructed based on these data.

The calculation process was implemented using JAGS 4.3.0 software. Four independent MCMC chains were set up, with each chain performing 1000 burn-in iterations to eliminate dependence on initial values, followed by 4000 sampling iterations. The convergence of the posterior distribution was ensured through diagnostics using the Gelman-Rubin statistic and autocorrelation function. The criterion for determining clinical significance was set as a threshold where the posterior probability that the population effect exceeded the MCID was >80%. Simultaneously, the type I error rate under the condition of a null effect was evaluated to ensure that the false-positive rate was controlled below the nominal level (α = 0.05). Systematic simulations revealed that when the sample size reached 14, the statistical power to detect the effect size was 86.0%, with a type I error rate of 2.0% at this point, which met the requirements of a target power of 80% and a controllable error rate.

### Statistical analysis plan

#### Data management

Analysis and reporting of the study results will follow the CONSORT guidelines, specifically the extension for *N*-of-1 trials (CENT 2015) [27]. The project management department of the Beijing Academy of Science and Technology serves as an independent data monitoring committee, supervising aspects of the trial, including participant recruitment, eligibility, randomization, compliance, and other critical elements. The committee will perform regular assessments and monitoring to ensure scientific integrity and ethical compliance throughout the trial. A designated study personnel member who is not involved in the data analysis will be responsible for regularly logging into the CGM platform (Sibionics Technology Co, Ltd) to check for any potential bias and misreported data. In case of significant violations, such as participants failing to comply with the trial requirements, being uncontactable for 3 consecutive days, or discontinuing participation due to health reasons, statistical analysis at the individual level will be precluded, and these cases will be excluded from meta-analysis at the population level.

#### Analysis of baseline data

Descriptive statistics will be used to summarize participants’ demographic characteristics. Continuous variables will be expressed as mean ± SD, whereas categorical variables will be reported as frequency and percentages. To compare baseline characteristics between participants who withdraw and those who complete the study, we will use *t* test for continuous variables and χ^2^ test for categorical variables.

#### Statistical hypotheses

At the individual level, the null hypothesis is that there is no difference in PBGP responses between any 2 staple foods for each participant, and the alternative hypothesis is that such differences exist. At the population level, the null hypothesis is that there is no difference in the average effect of PBGP induced by any 2 staple foods, and the alternative hypothesis is that such differences exist.

#### Individual-level N-of-1 trial analysis

To assess the PPGR to various staple foods at an individual level, Bayesian model will be performed. It is presupposed that individual observations of PBGP and iAUC_3h_ adhere to a normal distribution centered on each participant’s true average effect. The MCMC method will be used to estimate the posterior distribution of the parameters of interest. We will initiate 4 chains, with each executing 4000 iterations, culminating in a total of 16,000 sampling iterations. The Bayesian inference will be executed using the following equation for PBGP:

(1) $${\text{PBGP}}_{i}=\beta_{0}+\sum_{j}\beta_{j}\cdot {\text{staple}\;\text{food}}_{j}+\epsilon_{i}$$

in which the PBGP for participant *i* is modeled as the intercept, with the effect of each staple food *j* denoted as the intervention effect, and the random error for individual *i* modeled as a normal distribution with a mean of 0 and a large SD. Noninformative prior distributions will be incorporated into the model due to uncertainty regarding prior knowledge. Following the estimation of the posterior distribution of the effect of each staple food within the Bayesian model, the average value of the posterior distribution for differences between foods will be calculated, alongside the SE and 95% credible intervals. Standard procedures for warm-up periods, diagnostics for model convergence and stability, and residual checks will be applied. During the warm-up period, initial samples will be discarded to allow the chain to converge to a stationary distribution. The Gelman-Rubin test will be used to assess the convergence of the MCMC chains, with an *R*-hat value of <1.05, indicating that the chain has converged. The threshold for posterior probabilities will be set at 80% [28].

For a comprehensive evaluation of the PPGR of various staple foods among patients with diabetes, a weighted average response probability will be generated for each staple food. Using Bayesian models and sequentially using each staple food as a reference, we will estimate the posterior probability that the average difference between the reference food and the other 4 staple foods exceeds the MCID. As mentioned earlier, in this study, we define the MCID for PBGP as 0.78 mmol/L, and the MCID of iAUC_3h_ is 106.78 mmol/L·min, representing 10% of the difference between patients with T2DM and those with impaired fasting glucose, a difference that amounts to 1067.85 mmol/L·min [29].

For PBGP, the weighted glycemic response score (WGRS) for each staple food is calculated using the following equation:

(2) $${\text{WGRS}}_{i,j}=\frac{\sum_{k\neq j}{P\left({\Delta \text{PBGP}}_{i,j}>0.78\right)}_{k}\cdot \frac{1}{{\text{CV}}_{k}}}{\sum_{k\neq j}\frac{1}{{\text{CV}}_{k}}}$$

Equation 2 computes the sum of the posterior probability that, for participant *i*, the average difference between staple food j and other staple foods *k* exceeds the MCID of 0.78 mmol/L across multiple comparisons, divided by the coefficient of variation (CV) of each staple food *k*, which serves as a weight adjustment [30]. The result is then normalized by the sum of the reciprocals of the CVs. The WGRS of each staple food quantifies the probability of a clinically significant higher PBGP for that food across multiple comparisons. In the context of achieving a more stable glucose response, particularly in patients with diabetes, a staple food with a higher WGRS is less desirable.

#### Population-level N-of-1 trial analysis

For the population-level assessment of the PPGR to different staple foods, a Bayesian generalized linear mixed regression model will be used to aggregate the results from multiple *N*-of-1 trials at the population level, incorporating both between-individual and within-individual variations. Individual regression models will be linked using a random-effects model, assuming that the individual-specific regression coefficients follow a normal distribution centered on the population’s average coefficient. Noninformative priors will be used to define the variance distribution of the parameters, ensuring the robustness of the analysis. The threshold for posterior probabilities will be set at 80%. Additionally, the posterior probability of the mean differences exceeding the MCID will be evaluated.

#### Exploratory analysis

To investigate the role of gut microbiota in modulating glycemic responses to staple foods, the baseline metagenomic data will first undergo quality control filtering to exclude microbial features with low prevalence (species detection rate < 5%). Microbiota significantly associated with WGRS (*P* < 0.05) will then be preliminarily identified using Spearman correlation analysis, followed by the application of LASSO regression with 10-fold crossvalidation to compress high-dimensional data and identify core species. To further explore the relationship between gut microbiota characteristics and WGRS, a mixed-effects model will be constructed, with WGRS as the dependent variable, the screened microbiota features and covariates (e.g., age, gender, BMI, smoking, and alcohol consumption) as fixed effects and subject ID as a random intercept to account for individual variability. Additionally, the microbiota × staple food interaction term will be introduced to evaluate the potential regulatory effect of microbiota on staple food-specific glycemic responses, with multiple comparisons adjusted using the Benjamini-Hochberg false discovery rate correction at a significance threshold of <0.15.

### Patient and public involvement statement

In this study, patients and/or the public were not involved in the design, recruitment, or implementation of the experiment.

### Ethics approval and dissemination

The study protocol was approved by the Ethics Committee of the Inner Mongolia Nutrition Society. Our findings will be published in peer-reviewed journals or presented at international conferences. The full study protocol, anonymized participant-level data sets, and statistical code used to generate the results will be made available upon request from the corresponding author after the completion of the trial.

## Discussion

This study is designed to investigate the differences in PPGR among patients with diabetes consuming various staple foods using a series of *N*-of-1 trials. Real-time glucose fluctuations will be monitored via CGM as participants consume different staple foods. Bayesian multiple comparisons, in conjunction with a weighted averaging method, will be used to provide personalized dietary recommendations for each patient with diabetes, based on their WGRS on each staple food. Additionally, the study will explore the role of the gut microbiome in mediating glucose response variability. The results are expected to offer novel insights into personalized nutritional interventions for the management of diabetes.

There are significant differences in PPGR to different staple foods, primarily due to variations in their GI, which in turn affect individual PPGR. According to the 2021 International Glycemic Index and Glycemic Load Tables, there is substantial variation in the GI values among different staple foods [31]. In this study, 5 staple foods have been selected to represent either varying degrees of process on the same grain (refined rice, germ rice, and brown rice) or the different grains prepared using similar methods (rice noodles and pasta). The differing degrees of rice processing are known to influence the glycemic response by assessing GI. Refined rice, due to its rapid digestion and absorption, is a high GI food, resulting in a rapid rise in blood glucose concentrations postconsumption [32]. In contrast, brown rice, which retains more fiber, vitamins, and minerals, is digested more slowly, leading to less pronounced postprandial glucose fluctuations. Epidemiologic evidence has suggested that a high intake of refined rice was significantly linked to an increased incidence and mortality from T2DM, whereas intake of unrefined grains like brown rice was associated with reduced risk of T2DM [33,34]. A study using CGM to measure the 24-h glucose response to a high-fiber white rice diet among Asian Indian participants found that the average 24-h glucose response to high-fiber white rice was significantly lower than that to regular white rice [35]. A meta-analysis of randomized controlled trials indicated that pregerminated brown rice, in comparison with brown rice, had superior effects on lipid profiles and fasting blood glucose concentration [36]. Another meta-analysis reported that brown rice significantly influenced weight management in individuals with prediabetes and T2DM [37]. These findings highlight the importance of brown rice in controlling weight, reducing risk of diabetes, and improving overall metabolic health.

However, GI values of staple foods exhibit considerable variability among individuals, which poses a challenge to the universality of a standardized GI reference. Studies have shown that the SD of GI values within individuals and between individuals can reach 20%–25%, indicating that the same food can elicit significantly different blood glucose responses across different individuals. This suggests that the same food can elicit substantial differences in blood glucose responses across individuals. Therefore, this study aimed to address the variability in PPGR through an *N*-of-1 trial, exploring the specific impact of various staple foods on postprandial glucose load in patients with diabetes and identifying key factors that regulate these individual differences through gut microbiota analysis.

GI calculation typically relies on a relatively fixed blood glucose response curve, assuming data follows a normal distribution. However, this assumption is often not valid in practice. At the population level, GI involves ratios, which are often skewed or multimodal, rather than normally distributed, potentially affecting its reliability and validity. Unlike population-average GI, WGRS uses a Bayesian model to analyze the posterior probability of glucose fluctuations for each staple food, incorporating the CV of these differences as weights for a weighted average of the posterior probabilities. This approach integrates both the magnitude and stability of glucose fluctuations, offering a more accurate reflection of an individual’s blood glucose response to different staple foods in real-world settings.

Although CGM provides critical insights into dynamic glycemic responses, several technical considerations merit attention. First, the inherent physiological delay (5–15 min) between interstitial fluid glucose measurements and blood glucose measurements may cause a lag in detecting PBGP but does not systematically affect the relative differences in glycemic responses across interventions. Although the CGM devices used in this study showed slightly reduced accuracy in the hypoglycemic range, their reliability in the hyperglycemic range remained robust, ensuring dependable detection of primary end points in patients with T2DM [38]. Furthermore, as CGM accuracy declines over time, the replacement of sensors every 12 days in this study, coupled with a replicated crossover *N*-of-1 design, ensured a balanced distribution of residual drift across interventions. To account for potential individual-level random errors introduced by CGM, the Bayesian model incorporated individual-level variability through random effects, integrating uncertainty into posterior estimates. Although this protocol incorporates multiple rigorous measures to control potential confounding factors such as physical activity, dietary variations, and medication adherence, it is important to acknowledge that unquantified physical activity, chronic stress, or dietary changes on nontest days may introduce residual confounding effects, potentially influencing the interpretation of individual glucose responses. Future studies will leverage wearable devices for activity tracking, meal photography records and standardized dietary logs to further enhance precision.

This study uses an *N*-of-1 trial design to address the high individual heterogeneity in glycemic responses in diabetes management. Traditional research designs, based on population-average effects, often provide 1-size-fits-all dietary recommendations, overlooking the significant impact of biological differences, behavioral and lifestyle factors, and environmental influences on individual glycemic responses [39,40]. The *N*-of-1 design, through repeated and randomized within-individual crossover interventions, quantifies personalized responses of the same patient to different staple foods, enabling the formulation of dietary strategies based on the patient’s multidimensional characteristics. However, the broader application of the *N*-of-1 design requires balancing individualization with generalizability. Although this study aggregates individual data using Bayesian models to explore population trends, the generalizability of the conclusions remains limited by sample characteristics (e.g., sample specificity, staple food types, and processing methods) and external variables not fully controlled (e.g., physical activity, environmental stress). Future research could first use *N*-of-1 trials to identify response prototypes (e.g., microbiome-driven, insulin-sensitive, and lifestyle-dependent) and then validate subgroup-specific interventions through targeted randomized controlled trials, thereby balancing precision and scalability, and offering new insights for individualized interventions in diabetes management.

## Author contributions

The authors’ responsibilities were as follows – YW, YC, DM, JZ: performed investigation, methodology, formal analysis, and data curation and wrote the original draft; CL, JD, YG, SZ, BL: performed investigation, reviewed and edited the manuscript, and administered the project; YW, YC: curated the data; DM, JZ: conceptualized and supervised the study and reviewed and edited the manuscript; and all authors: have read and agreed to the published version of the manuscript.

## Data availability

The datasets used and/or analyzed during the current study are available from the corresponding author upon reasonable request.

## Funding

This work was supported by the Financial Program of BJAST (No.24CA008, No.25CA008)..

## Conflict of interest

The authors report no conflicts of interest.
