# Supplementary material for: Protocol of a Series of N-of-1 trials for Exploring Personalized Blood Glucose Responses to Different Staple Foods in Patients with Diabetes
Source: Curr Dev Nutr. 2025 Oct 13;9(11):107575. doi: 10.1016/j.cdnut.2025.107575 (PMC12639426; doi:10.1016/j.cdnut.2025.107575)
Supplement: Multimedia component 1 [file mmc1.docx]

**Title: Protocol of a series of N-of-1 trials for exploring personalized blood glucose responses to different staple foods in diabetic patients**

1st author: Yuyang Wang

**Informed Consent Form · Informed Notification Page**

Dear volunteers,

You will be invited to participate in a study of personalized differences in blood glucose response to different staple foods. This study protocol has been reviewed by the Ethics Committee of Inner Mongolia Nutrition Society and agreed to conduct clinical research.

Before you decide whether to participate in this study, please read the following as carefully as possible. It can help you understand the study and why it was conducted, the procedures and duration of the study, the benefits, risks and discomfort you may have had after participating in the study. If you wish, you can also discuss with your relatives and friends, or ask a doctor or researcher to give an explanation to help you make your decision.

We will send out the specific solicitation content of this study to the society through poster publicity and network publicity, and inform the volunteers of the specific participation time, precautions and related details of this study by SMS or phone call.

1. **Research background and study objectives**

**1.1 Research Background**

Diabetes is a metabolic disease characterized by hyperglycemia, and type 2 diabetes is the most dominant type, accounting for 90% of the total number of diabetic patients, and its incidence increases with age. According to the 2019 report of the International Diabetes Federation, about 460 million people suffer from diabetes worldwide, and about 120 million people have diabetes in China, with the largest number of diabetic patients in the world. The prevention and treatment of type 2 diabetes requires a series of comprehensive measures such as health education, diet control, exercise, blood glucose monitoring and drug therapy. Among them, diet control is the basis of blood control treatment, its importance is increasingly highlighted, is considered should throughout diabetes management, and carbohydrates occupies a large proportion in human diet, has a direct relationship with blood sugar fluctuations, so the focus of diabetic diet control is the reasonable intake of carbohydrates.

In the diet of Chinese residents, the common staple food includes rice, wheat, corn, millet, oats, etc., which is the main source of carbohydrates. Changes in the carbohydrate composition and content of these staple foods may affect the blood glucose response after staple food intake. Therefore, the selection of the quantity and types of staple foods through scientific methods can strengthen the regulation of blood glucose homeostasis and promote the prevention and treatment of diabetes.

This study intends to use a single-case randomized controlled experiment to study the personalized differences in blood glucose response to different staple foods in diabetic subjects.

1.2 Study Purpose

In this study, the continuous blood glucose monitor will be used to monitor the subjects&#; daily blood glucose, so as to obtain individual blood glucose response to different staple foods, and evaluate the selected staple foods, so as to help diabetic patients to better choose the appropriate staple food and provide suggestions for the daily diet management and prevention of diabetes.

1.3 Overall design

1. With a continuous blood glucose monitor, the blood glucose response to different staple foods.

2 . It is helpful to evaluate the selected staple food, help diabetes patients to better choose the appropriate staple food, and provide suggestions for the daily diet management and prevention of diabetes.

1.4 Subject inclusion

1. Healthy people and type 2 diabetes (only patients with diabetes through lifestyle intervention, or only patients using monotherapy for blood glucose control);
2. Adults, aged under the age of 70;
3. Those with no difficulty in chewing;
4. No lactose intolerance, egg allergy, etc.;

## Signed the informed consent form.

**1.3 Number of participating units and included volunteers**

The research was conducted by the Institute of Biotechnology and Health, Beijing Institute of Science and Technology. Beijing institute of science and technology is the only large multidisciplinary research institutions, with 27 hospital units, urban safety and management, urban environment and ecological protection, &quot;advanced&quot; technology and industry, biological medicine and health, science and technology innovation, a think-tank, science popularization and culture communication and so on six big key areas of scientific research institutions, has three national (ministry) level key laboratory, 18 Beijing municipal key laboratory / engineering center. With the second generation of gene sequencing laboratory, molecular biology laboratory, microbiology laboratory, clinical laboratory, functional evaluation laboratory, milk function laboratory high performance server room, equipped with Hiseq2500 high-throughput gene sequencing instrument, Miseq gene sequencing instrument, ABI 7500fast real-time PCR, liquid phase high resolution mass spectrometry equipment, can meet the project for in vitro fermentation, breast milk glycopeptide detection, peptide detection after digestion, intestinal flora sequencing requirements.

This study is planned to enroll 20 subjects, of both sexes.

**2. Who should not take part in the study**

1. Pregnant women, lactating mothers or pregnant people

2. Severe metabolic diseases, digestive system disorders, and psychiatric disorders

3. Special dietary structure (such as ketogenic diet, low-carb diet, etc.)

4. Frequent business trips

## 5. People with easy skin allergy (such as urticaria)

**3. What will you need to do if you participate in the study?**

1. Provide basic information about the subjects: age, sex, height, weight, waist circumference, hip circumference, blood pressure, diabetes history, history of medication taking, other diseases, surgery history, etc.

2. Participated in the physical examination organized by the research group, and collected saliva, blood, urine, stool samples, etc.

3. During the implementation of the project, wear the continuous blood glucose monitor, eat the breakfast of the 6 different main foods as required by the research group, and make meal records and exercise records.

If you volunteer to participate in the study, you will proceed as follows:

(1) Participate in the volunteer training and explain the content of the informed consent form.

(2) Complete the basic information collection and the initial physical examination.

(3) Wear a continuous blood glucose monitor according to the requirements of the project, and record the blood glucose value.

(4) Eat different staple foods in turn, and record the diet and exercise conditions together.

**4. Possible benefits of participation in the study**

You can get RMB 200 or an equivalent gift for participating in this study. Your participation will help you understand the difference in blood glucose response after eating different staple foods, which will help your diet management and benefit the society. At the same time, the relevant information about the metabolism of intestinal microflora can also be obtained.

**5. Possible adverse reactions, risks and discomfort, and inconvenience of participating in the study**

This trial is a food intervention, no adverse product intervention, only saliva, blood, urine, stool samples collection, will not bring health risks.

If you are abnormalities during the study, or your willingness to participate changes, please contact the researcher.

Investigation takes up some of your time and will not cause you trouble or inconvenience.

**Vi. Related expenses**

The investigation cost of the trial arrangement will be borne by the study party.

If you are injured by participating in this study, we will provide the necessary medical measures. According to the relevant laws and regulations in China, the researcher will bear the corresponding medical expenses and provide the corresponding economic compensation.

The treatment and examinations required for your other diseases will not be free of charge.

Since the participants are all working age, they may delay work due to the trial and will give appropriate compensation for missing work, and transportation subsidies will be provided to the participants to participate in the trial.

**7. Is the personal information kept confidential?**

If you decide to participate in this study, your participation in the study and your personal data in the study are kept confidential. Any information that will identify you will not be disclosed to members outside the study team until your permission. All study members and study related parties will keep your identity confidential as required. Your file will be kept in a locked file cabinet for researcher access only. To ensure that the study is conducted in accordance with the regulations, if necessary, members of the government administration, research institution or ethics committee can access your personal data at the study site as required. No personal information of you will be disclosed on the results of this study.

According to medical research ethics, in addition to personal privacy information, trial data will be available for public inquiry and sharing, and it will be limited to web-based electronic databases, ensuring that no personal privacy information will be leaked.

**8. How do you get more information?**

You can ask any questions about this study at any time and answer them accordingly.

Your investigator will promptly notify you of any important new information during the study that may affect your willingness to continue participating in the study.

**9. You can voluntarily choose to participate in the study and withdraw from the study**

Whether you will participate in the study is entirely dependent on your wishes. You may refuse to participate in the study or withdraw from the study at any time during the study. This will not affect your relationship with the researcher or the loss of your other benefits.

For your best interest, the investigator may discontinue this study at any time during the course of the study.

If you withdraw from the study for any reason, you may be asked about your use of the test samples. You may also be required to have a laboratory and physical examination if deemed necessary.

**10. What should I do now?**

Whether or not to participate in this study is up to you (and your family members).

Ask the researcher for questions whenever possible before you make the decision to participate in the study.

Thank you for reading the above materials. If you decide to participate in this study, please tell the researcher that he / she will arrange everything for you about the study. Please keep this information.

Informed Consent · Consignature page

**Clinical study project Name: Study on personalized differences in blood glucose response to different staple foods**

**Project contractor: Institute of Biotechnology and Health, Beijing Institute of Science and Technology**

**Consent statement**

I have read the above introduction to this study and have the opportunity to discuss and ask questions with the researcher on this study. All the questions I have raised have been answered satisfactorily.

I am aware of the risks and benefits that may arise from participating in this study. I know that participation in the study is voluntary, and I confirm that I have enough time to consider this, and I understand that:

(1) I can consult the researchers for more information at any time.

(2) I can withdraw from this study at any time without discrimination or retaliation, and my treatment and interests will not be affected.

I agree with the ethics committee of the drug Authority or the sponsor representative to access my study data.

I will obtain a copy of the signed and dated informed consent form.

Finally, I decided to agree to participate in this study and promised to follow the study procedures as much as possible.

Subject Signature: Contact phone number:

________ Year _________ month _________ day

I confirm that the volunteers have explained the details of this trial, including its authority and the possible benefits and risks, and have given them a copy of the signed informed consent form.

Investigator Signature: Contact Number:

_______ Year _________ month _________ day
